# A Tunnel Crack Segmentation and Recognition Algorithm Using SPGD-and-Generative Adversarial Network Fusion

**DOI:** 10.3390/s25082381

**Published:** 2025-04-09

**Authors:** Wei Sun, Xiaohu Liu, Zhiyong Lei

**Affiliations:** 1 School of Mechatronic Engineering, Xi’an Technological University, Xi’an 710021, China; gemlei@xatu.edu.cn; 2 School of Mechanical Engineering, Shaanxi University of Technology, Hanzhong 723001, China; 3 Trine Engineering Institute, Shaanxi University of Technology, Hanzhong 723001, China; lxh@snut.edu.cn; 4 School of Electronic Information Engineering, Xi’an Technological University, Xi’an 710021, China

**Keywords:** tunnel crack, stochastic parallel gradient descent (SPGD), generation adversarial network (GAN), image segmentation

## Abstract

In order to improve the recognition ability of tunnel cracks in the UAV platform with a vision imaging system in the UAV platform with a vision imaging system, this paper proposes a tunnel crack segmentation algorithm using SPGD-and-generative adversarial network fusion. The SPGD algorithm can enhance the detail and edge information of a tunnel crack image, which improves the clarity of the tunnel crack image. The new generative adversarial network (GAN) is designed by using an improved U-Net generator and full convolutional network (FCN) discriminator to form a new network; the improved generative adversarial network can effectively segment tunnel crack images after stochastic parallel gradient descent (SPGD) algorithm processing, especially the texture feature extraction and segmentation of small tunnel cracks, which can improve the rate of recognition of tunnel cracks. Based on collected tunnel crack image data, we selected 12 typical tunnel crack images and verified the rationality and advanced nature of the proposed recognition algorithm by comparing it with other recognition methods. The results show that the recognition rate of the proposed tunnel crack recognition algorithm was significantly improved.

## 1. Introduction

Tunnel crack detection plays a vital role in the safe operation of tunnel roads. Tunnel cracks not only significantly reduce the service life of a tunnel but may also lead to structural instability or even collapse, posing a great threat to traffic safety [1]. Therefore, crack detection has become a key means to prevent and eliminate such disasters. However, due to the particularity of the tunnel environment, crack detection faces many challenges. The geological conditions of tunnels are complex and changeable. Fluctuations in groundwater levels and temperature changes cause irregular crack textures [2]. This irregularity makes it difficult to identify cracks as intuitively as conventional large cracks. Among them, fine-texture cracks are difficult to detect using conventional methods due to their subtle and irregular nature. Fine-texture cracks may be hidden under the surface of the tunnel lining or blend with the texture and color of the lining, making them particularly challenging to identify. However, over time, these cracks gradually expand, posing a serious threat to the structural safety of the tunnel. Additionally, the significant variations in illumination conditions, dust, environmental noise, and the brightness of tunnel surface further complicate the detection process [3,4]. These factors make it difficult to establish a unified method for tunnel crack detection, resulting in low detection efficiency.

To address these deficiencies, tunnel crack detection technology based on UAVs (unmanned aerial vehicles) has emerged. UAVs can be equipped with cameras and imaging equipment, utilizing their flight flexibility and storage modules to quickly obtain image data inside a tunnel. Through datasets, combined with advanced image recognition algorithms, cracks can be effectively identified and analyzed. However, when a UAV platform with a vision imaging system is used to collect tunnel images, because of uncertain factors such as shaking in the work of the UAV, the quality of the images collected by the vision system is often low, resulting in the tunnel crack texture features not being obvious, which increases the difficulty of tunnel crack identification.

At present, many researchers have conducted much research on tunnel crack identification. For example, He et al. proposed an improved YOLOv7 algorithm [5,6], which optimizes the network structure and introduces an attention mechanism, significantly improving the accuracy and real-time performance of crack detection. A real-time crack detection algorithm based on GRU-CNN is used to extract key information about cracks through frequency-domain processing. Li et al. [7] proposed an improved Canny operator to optimize the edge detection algorithm and enhanced the recognition ability of fine cracks. Huang et al. [8] studied a crack detection algorithm based on connected domains. The piecewise SPGD algorithm demonstrates transferable value for image segmentation tasks despite its original design purpose. The algorithm dynamically adjusts network parameters during training to improve model robustness against noise and uneven illumination [9]. In view of the problem that the number of pavement crack images cannot meet basic needs for deep learning, according to using generative adversary networks to expand datasets, a pavement segmentation algorithm based on the U-Net network was proposed [10]. The Grack-GAN enhancement method based on GANs solves the problem of uneven sample distribution in pavement crack recognition tasks [11]. Based on GANs, an image dataset of bridge cracks was expanded, and the overfitting problem of the model was alleviated by enriching the samples [12].

In order to improve the efficiency of traditional tunnel crack image recognition, many researchers have carried out targeted improvement studies based on actual tunnel crack images in different environments. Bao et al. [13] proposed a modified YOLO (M-YOLO) algorithm based on YOLOv8 to identify cracks in tunnels; this algorithm adopts full-dimensional dynamic convolution to replace the traditional convolution module, remarkably improving detection accuracy and avoiding model parameter expansion. Kuang et al. [14] proposed a new crack segmentation method, CrackViT, based on a lightweight Transformer. Pan et al. [15] developed a novel approach using EDeepLab to achieve better precision detection and segmentation of lining surface cracks; EDeepLab improves upon the original DeepLabV3+ framework by replacing its backbone network with the optimized lightweight EfficientNetV2. Li et al. [16] proposed a novel dual-view snake Unet (DSUnet) model, which integrates a hybrid snake cascading (HSC) module and a Haar wavelet down-sampling (HWD) operation; the HSC module enhances the network’s capability of extracting tunnel lining cracks by synergistically combining features derived from standard convolutions and bidirectional dynamic snake convolutions, thereby capturing intricate geometric and contextual information. These methods can accurately detect cracks in cases where the cracks are clear and have good effects; when the quality of images collected by the vision system is low, many fine-texture cracks often lose their characteristics during recognition.

To improve the recognition ability of tunnel cracks in a UAV platform with a vision imaging system, a UAV-based visual inspection system consists of an unmanned aerial vehicle equipped with imaging components including a high-speed camera, onboard storage module, and data processing unit. During operation, the drone flies at predetermined altitudes within the tunnel to capture high-resolution surface images under controlled distance parameters. These acquired images are initially stored in the drone’s onboard memory before being transmitted via a high-speed data link to the ground processing station. The ground-based computer system subsequently performs crack detection and identification through specialized image processing algorithms with the received image sequences. Based on this research object and background, we studied the fusion of a stochastic parallel gradient descent (SPGD) algorithm and generation adversarial network (GAN) to identify tunnel cracks.

The main highlights of this article are as follows:

(1) We propose a tunnel crack segmentation algorithm based on the fusion of a stochastic parallel gradient descent (SPGD) algorithm and generation adversarial network (GAN). The SPGD algorithm can enhance the detail and edge information of a tunnel crack image, which improves the clarity of the tunnel crack image.
(2) We design a new generative adversarial network (GAN) by using an improved U-Net generator and full convolutional network (FCN) discriminator to form a new network; the improved generative adversarial network can effectively segment tunnel crack images after stochastic parallel gradient descent (SPGD) algorithm processing, especially the texture feature extraction and segmentation of small tunnel cracks, which can improve the rate of recognition of tunnel cracks.

This paper is structured as follows: Section 2 describes the dataset preparation. Section 3 presents the tunnel crack segmentation algorithm based on SPGD-and-GAN fusion. Section 4 details the experiment and analysis. Finally, the conclusion is drawn in Section 5.

## 2. Dataset Preparation

**Figure 1:**
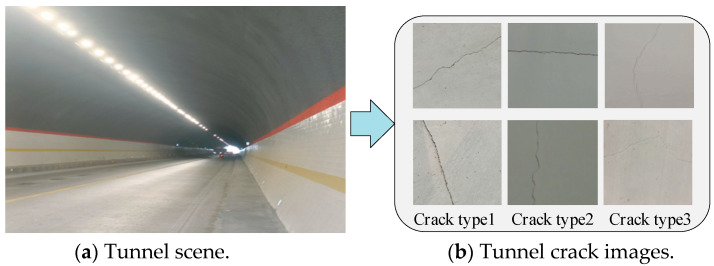
The tunnel scene and tunnel crack images.

The quality of a segmentation dataset significantly impacts the performance of a segmentation model and its engineering applications. In this study, tunnel data were collected using unmanned aerial vehicles (UAVs), and Figure 1 is the tunnel scene and tunnel crack images; Figure 1a is the tunnel scene, and Figure 1b is the collected tunnel crack images.

**Figure 2:**
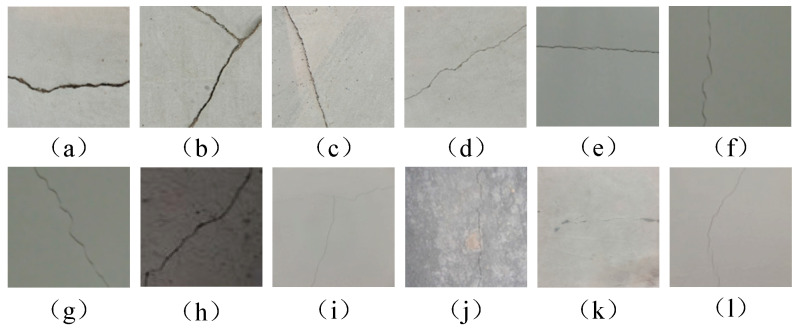
Various types of tunnel crack image data. (**a**–**d**) are crack images under normal lighting under; (**e**–**h**) are crack images under low-illumination conditions; (**i**–**l**) are fine-textured crack images.

From the images collected by the camera equipped on the UAV, 1000 three-channel crack images were selected to construct the segmentation dataset. Firstly, all the pictures were preprocessed, and the pictures were converted into 1024 × 1024 pixels. The actual size of each pixel was 0.976 mm × 0.976 mm, and we performed manual semantic annotation and length and width information labeling, storing the data in YOLO format while including target object information. At the same time, we defined cracks with widths less than or equal to one millimeter as fine-texture cracks and cracks greater than one millimeter as conventional cracks. Various types of crack image data are shown in Figure 2.

Figure 2a–d are crack images under normal lighting. The cracks are clear with sharp edges and distinct texture details. They have high contrast and minimal background interference, making crack information easier to identify. Figure 2e–h are crack images under low-illumination conditions. These have low contrast and high noise, making crack features hard to distinguish. Cracks may be masked by background noise, blurring their edges and textures. Figure 2i–l are fine-textured crack images, where the cracks are small, irregularly textured, and blend with the surrounding texture and color. This makes the cracks unclear and difficult to identify.

## 3. Tunnel Crack Segmentation Algorithm Based on SPGD-and-GAN Fusion

**Figure 3:**
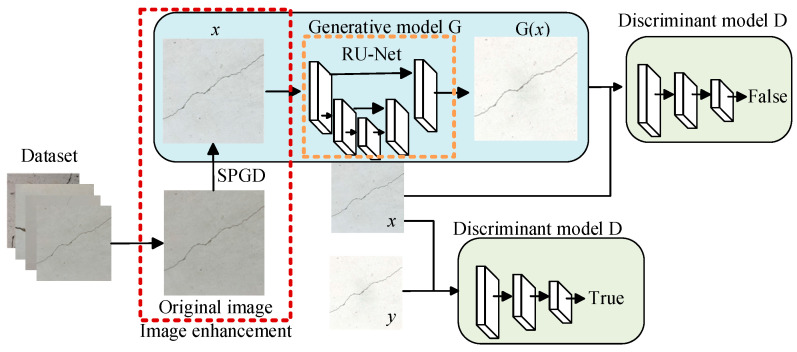
The structure and processing flow of the algorithm based on SPGD and GAN fusion.

To address issues such as image blurring caused by UAV vibration, uneven illumination, and indistinct crack features in tunnel images captured by UAV-mounted cameras, this paper employed the SPGD algorithm to enhance the details of tunnel crack images. The structure and processing flow of the algorithm based on SPGD-and-GAN fusion is shown in Figure 3.

The tunnel crack segmentation network model proposed in this paper is primarily based on the GAN framework, which consists of a generative model, G, and a discriminative model, D. The generative model G learns from the crack image data in the dataset to generate crack segmentation results. Simultaneously, the image data are combined with the generated crack segmentation results to serve as the negative sample input for the discriminative model. The input of the discriminative model D consists of positive samples, which are composed of crack images from the dataset and their corresponding manually labeled crack results. The training objective of the generative model G is to produce crack segmentation images that closely match the annotation results of the discriminative model D, thereby deceiving the discriminator and making it difficult to distinguish between the generated crack segmentation results and the real annotated results. The discriminative model D plays a critical role in the entire crack segmentation process. By comparing the input negative sample results with the positive sample results, the model parameters are optimized to make the system’s judgment of the positive samples approach 1. Through this adversarial learning mechanism, Crack-GAN can effectively improve the accuracy and robustness of crack segmentation.

The generative model G and the discriminative model D are trained through alternating iterations. The input of the discriminative model D includes two types of samples: one is the combination of a crack image and the manual annotation result (positive sample), and the other is the combination of a crack image and the segmentation result generated by the generative model G (negative sample). By leveraging the discriminative ability of D, the distinction between these two samples is achieved. During the training process, the generative model G uses the judgment results of the discriminative model D as the optimization goal. It receives feedback from D to propagate errors and updates its parameters accordingly. As training progresses, the output of G gradually approximates the positive samples in D, ultimately achieving the optimization segmentation results.

### 3.1. Image Enhancement Based on SPGD Algorithm

**Figure 4:**
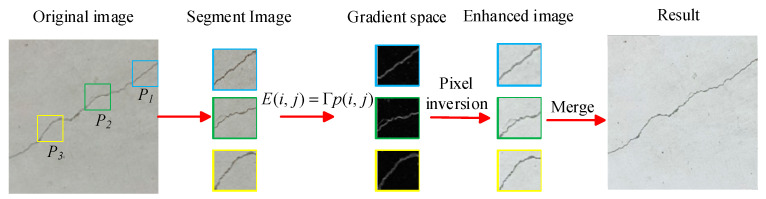
The image enhancement process using the SPGD algorithm.

Fine-textured cracks in tunnels are small in size and irregular, and cracks of this type in images are difficult to identify by conventional detection methods. As time goes by, these cracks gradually expand, posing a serious threat to the structural safety of tunnels. In this paper, the SPGD algorithm was used to optimize the crack details by enhancing the gradient amplitude and adjusting the gradient direction. While maintaining the overall structure of an image, the details of the cracks in the image could also be enhanced [17,18,19]; the enhancement process of the SPGD algorithm is shown in Figure 4.

To achieve the precise enhancement of crack features, the global crack–pixel ratio of an entire image is first calculated as a density benchmark. A sliding window is then employed to evaluate local crack densities. The sub-block size is dynamically adjusted based on discrepancies between local and global densities, with iterative optimization ensuring balanced crack distribution across all sub-blocks.

The tunnel crack image is partitioned into multiple sub-blocks, denoted as $P=\{p_{1},p_{2},\ldots ,p_{m}\}$, where *m* represents the number of regions in the partitioned crack image. A random tunnel crack sub-image block is selected from the partitioned crack image set *P*, and its gradient function is calculated using a gradient transformation operator. The image block is then transformed into gradient space, with pixel values denoted as p(i,j). The gradient function can be expressed by Formula (1).

(1) $$E(i,j)=\Gamma p(i,j)=\left[\begin{array}{c} F_{i}\\ F_{j} \end{array}\right],$$

where Γ represents the gradient transformation operator, and $F_{i}$ and $F_{j}$ represent the partial derivatives of p(i,j) with respect to *i* and *j*, respectively. Based on this, the desired gradient function is calculated using a transformation function and three constants, α, β, and γ, whose values range between 0 and 1. Formula (2) is the desired gradient function, and Formula (3) is the transformation function.

(2) G(i,j)=f(i,j)∗E(i,j),

(3) $$f(\begin{array}{c} i,j)=∥E(\begin{array}{c} i,j) \end{array}\cdot \alpha ∥-\beta^{-∥E(\begin{array}{c} i,j) \end{array}{∥}^{\gamma }} \end{array},$$

where α, β, and γ are constants with values ranging from 0 to 1. To optimize the reconstruction results, the least-squares method is used to minimize the error between the desired gradient function G(i,j) and the original gradient function $E(\begin{array}{c} i,j) \end{array}$ [20,21,22,23,24].

The initial values of α, β, and γ are randomly selected, and the parameters are updated iteratively in the negative-gradient direction until the stopping condition is met, thereby obtaining the optimal parameters. Finally, these optimal parameters are used to construct the transformation function, which is applied to the pixels in the gradient space. The transformed pixels are then inversely mapped back to the original space. The above process is repeated for all sub-blocks, and the enhanced sub-blocks are fused to obtain the complete enhanced image.

### 3.2. Improved U-Net Generator Design

**Figure 5:**
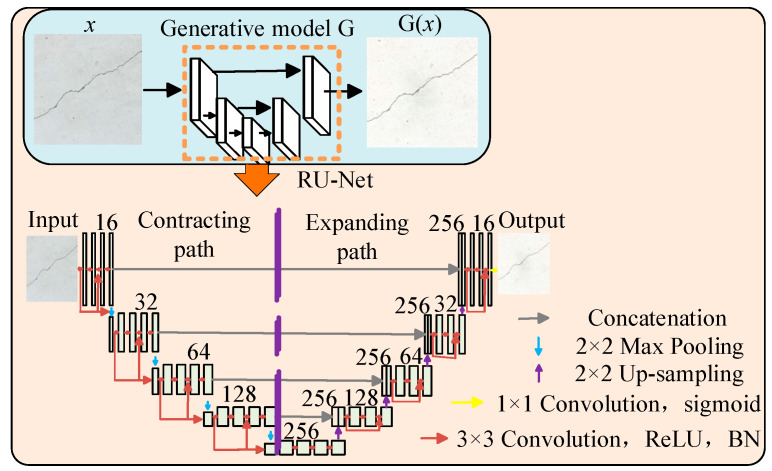
RU-Net network structure.

The generative model extracts feature from crack images and produces segmentation results of the same size as the original crack images. The crack segmentation model takes the complete crack image as an input, offering advantages in terms of structural distribution. Specifically, it captures more local information features within smaller receptive fields. To achieve the effective feature extraction of crack images under complex backgrounds, a RU-Net model was designed by leveraging the characteristics of residual networks, as illustrated in Figure 5.

The network model consists of an encoder with four network layers, connected through max pooling convolutions. The encoder includes four residual modules, each composed of convolutional layers and shortcut connections. Similarly, the decoder is structured with four network layers connected by 2 × 2 convolutional layers, containing four residual modules built from convolutional layers. To achieve more efficient network training and prevent overfitting, the number of convolutional kernels in each layer is reduced [25,26]. Additionally, batch normalization layers and ReLU activation functions are incorporated after the convolutional layers.

### 3.3. Discriminator Design

**Figure 6:**
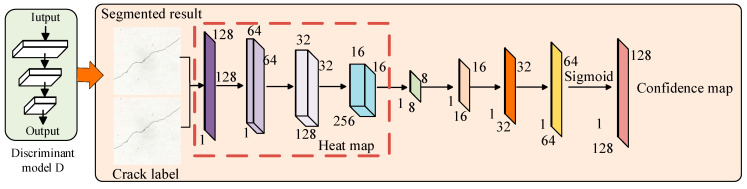
A discriminator network structure.

To implement semi-supervised adversarial learning for crack image segmentation, this paper designed the discriminator network as an FCN, enabling it to output a confidence map of the same size as the input image. Unlike traditional GANs [27,28,29,30], where the discriminator outputs a single true/false value, the proposed discriminator network generates a two-dimensional confidence map instead of a single label. This confidence map reflects the probability of each pixel in the input segmentation result being derived from expert segmentation, i.e., the likelihood of each pixel being segmented according to the ground truth. The structure of the discriminator network is illustrated in Figure 6.

The discriminator network structure consists of four convolutional layers and four bilinear interpolation layers. Each convolutional layer employs a 4 × 4 kernel, with channel numbers set to 64, 128, 256, and 1, respectively. Additionally, mean-variance normalization is utilized to maintain a uniform distribution of feature vectors, thereby accelerating the convergence of the convolutional network. The first four convolutional layers generate a two-dimensional heatmap, where each pixel value represents the likelihood of a perfect match with the label. Subsequently, four bilinear interpolation layers are used to up-sample the heatmap to the same resolution as the input segmentation result and the expert label. Finally, a confidence map is generated through the sigmoid activation function.

### 3.4. GAN Training

**Figure 7:**
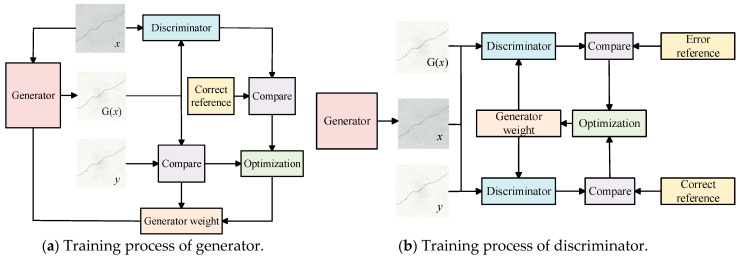
The training process of the generator and discriminator.

During the training process, the weights of both the generator and the discriminator are continuously adjusted to ensure that the crack images segmented by the generator closely approximate the manually labeled crack images, thereby improving the recognition accuracy of the discriminator. The training process of generator and discriminator is illustrated in Figure 7.

The adjustment of the generator’s weight is performed through two approaches. First, the weight is adjusted by comparing the manually annotated crack label image *y* with the crack image segmented by the generator, aiming to minimize the difference between them. Second, the manually annotated crack label image *y* and the crack image generated by the generator are input into the discriminator, and the weight is adjusted to make the discriminator’s output approach 1.

To improve the recognition accuracy of the discriminator, the weight is adjusted in two ways: first, by inputting the original crack image *x* and the manually annotated crack label image *y* into the discriminator and continuously adjusting the discriminator’s weight to make the output D(x,y) approach 1; second, by inputting the original crack image *x* and the crack image G(x) generated by the generator into the discriminator and continuously adjusting the discriminator’s weight to make the output D[G(x),y] approach 0.

### 3.5. Loss Function

In the generation model, the mean absolute error (MAE) is used to determine the mean absolute error between the target variable and the prediction variable [31]. The loss range is [0,∞]. The MAE determines the average size of the error in the prediction without considering the direction of the error. It has better robustness to discrete points. Formula (4) is its calculation function.

(4) $$MAE(y_{i},{\hat{y}}_{i})=\frac{1}{n}\sum_{i=1}^{n}|y_{i}-{\hat{y}}_{i}|,$$

In the discriminant model, the mean square error (MSE) is used to determine the degree of loss according to the sum of squares of the distance between the target variable and the predicted value. It is easier to solve. Formula (5) is its calculation function [32,33,34].

(5) $$MSE(y_{i},{\hat{y}}_{i})=\frac{1}{n}\sum_{i=1}^{n}{\left(y_{i}-{\hat{y}}_{i}\right)}^{2},$$

where $x_{k}$ is the actual value of the *k*-th pixel label, $y_{k}$ is the predicted value of the *k*-th pixel label, and *n* represents the sample size.

## 4. Experiment and Analysis

### 4.1. Test Environment

To validate the effectiveness of the proposed algorithm, we employed a multi-source data fusion approach, integrating a self-collected UAV dataset (1500 images) with authoritative public tunnel datasets—the Crack dataset (1000 tunnel crack images) and Crack Forest Dataset (CFD) (500 complex-background crack images)—to construct a comprehensive multi-scenario dataset comprising 3000 high-quality crack images. In this paper, based on the 3000 images containing cracks selected from captured images and authoritative public tunnel datasets, to facilitate model training, the window sliding algorithm was utilized to cut the dataset according to the size of 512 × 512 pixels; then, through horizontal flipping, vertical flipping, rotation transformation, and other enhanced data processing methods, 10,000 images of 512 × 512 pixels were obtained. In the processing, the dataset was divided into a training set and test set according to 8:1.

The experimental environment is shown in Table 1. The Adam optimizer was used for iterative training. The training batch number was 8, the number of training rounds was 200, and the initial learning rate was 0.0001. In the training process, data enhancement methods such as random flipping, random scaling, and illumination changes were used to increase the number of training data, solve the problem of overfitting and sample imbalance, and improve the generalization ability of the model.

### 4.2. Evaluation Indicators

To validate the accuracy of the proposed model and assess whether it meets the requirements for real-time recognition, this paper employed evaluation metrics including pixel accuracy (PA), precision (P), recall (R), F1 score, IoU, and mAP@50. PA provides a quick assessment of the model’s overall performance in classifying crack and non-crack pixels in crack segmentation, measuring the overall classification accuracy across all pixels and reflecting the model’s effectiveness in classifying the entire image [35,36,37,38,39,40]. P evaluates the accuracy of the model’s crack prediction, indicating the reliability of the model ’s recognition of cracks, that is, the proportion of predicted crack pixels to actual cracks. R measures the model’s ability to detect cracks and reflects the model’s coverage of actual crack pixels, that is, how many real cracks were detected. The high recall rate indicates that the model rarely missed the actual crack pixels. The four pixels evaluation categories in the crack segmentation results are shown in Table 2.

In Table 2, TP (True Positive) represents the number of pixels correctly predicted as cracks by the model, FP (False Positive) denotes the number of pixels incorrectly identified as cracks by the model when they were actually non-cracks, FN (False Negative) indicates the number of pixels incorrectly identified as non-cracks by the model when they were actually cracks, and TN (True Negative) represents the number of pixels correctly identified as non-cracks.

The calculation formula of pixel accuracy is

(6) $$PA=\frac{TP+TN}{TP+FP+FN+TN},$$

The calculation formula of the precision rate is

(7) $$P=\frac{TP}{TP+FP},$$

The calculation formula of the recall rate is

(8) $$R=\frac{TP}{TP+FN},$$

The calculation formula of the *F*1 value is

(9) $$F1=\frac{2\times P\times R}{P+R},$$

The calculation formula of IoU is

(10) $$IoU=\frac{TP}{TP+FP+FN},$$

The calculation formula of mAP@50 is

(11) $$mAP@50=\frac{1}{N}\sum_{i=1}^{N}AP_{i}{|}_{IoU=0.5},$$

### 4.3. Image Enhancement Effect

To address the problem of unclear crack images, we adopted the SPGD algorithm to adjust the gradient magnitude and direction of images in a stochastic parallel manner, thereby significantly enhancing the visibility of key features such as cracks while maintaining the overall image structure. A grid search method was used to systematically optimize three key parameters of the SPGD algorithm; among these parameters were the gradient magnitude enhancement coefficient α∈[0.1,0.9], the direction correction weight β∈[0.1,0.9], and the structure preservation coefficient γ∈[0.1,0.9], respectively.

**Figure 8:**
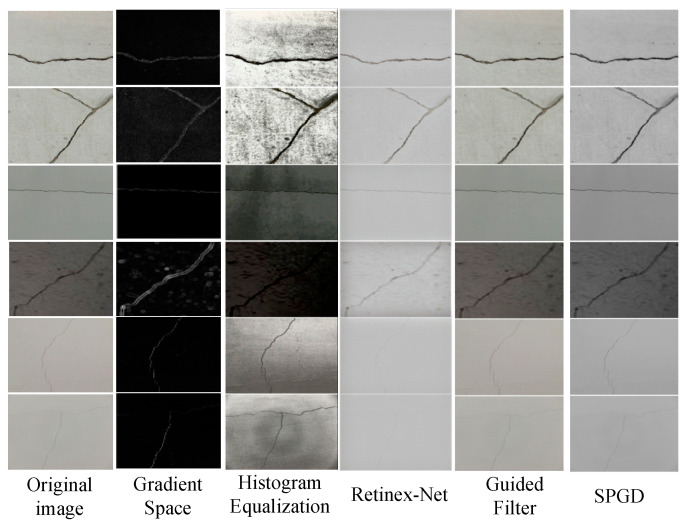
Image enhancement effect of different algorithms.

The three curves correspond to α, β, and γ, respectively. The experimental results demonstrate that the optimal parameter combination (α = 0.7; β = 0.3; γ = 0.5) achieved an IoU of 73.8% on the validation set. Among these parameters, α exhibited the most significant impact on the results, while β and γ showed relatively robust performance. Bayesian optimization verified the reliability of these findings. This specific combination effectively enhanced faint crack features while preserving texture authenticity and maintaining a balance between detail enhancement and noise suppression. Six crack images containing various categories are selected from the comprehensive multi-scenario datasets and a comparison of enhancement effects is shown in Figure 8.

From the results of Figure 8, we can find that the performance of different image enhancement algorithms in crack feature extraction and recognition showed significant differences. Histogram equalization could enhance the overall contrast of an image, making cracks more distinct. However, it tended to amplify interfering signals, which led to suboptimal performance in the recognition of low-contrast cracks. Retinex-Net, through multi-scale detail fusion and background compensation, significantly enhanced the details of cracks and performed well under complex lighting conditions. Yet, it may have introduced halo effects and did not work as well for fine cracks. Guided filtering could remove noise while maintaining the clarity of crack edges, but its effectiveness depended on the quality of the guiding image. The SPGD image enhancement algorithm sharpened crack edges and highlighted details, performing particularly well in the recognition of fine-textured cracks. By comparing the application effects of these algorithms on different crack images, it can be concluded that the SPGD algorithm outperformed others in handling fine-textured cracks under complex backgrounds and noise interference.

### 4.4. Ablation Experiment

**Figure 9:**
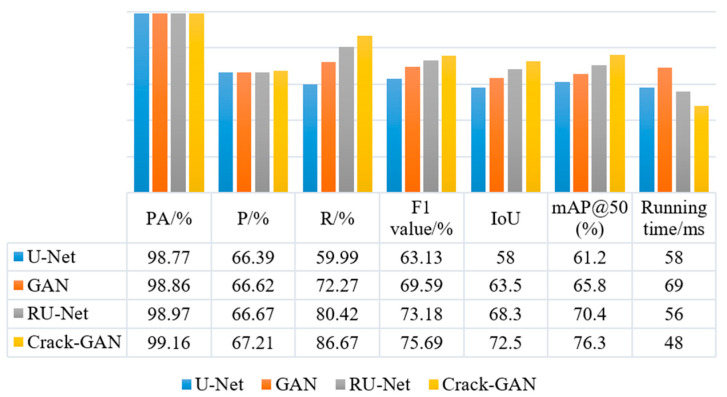
Ablation results of different network modules.

To further verify the effectiveness of the introduced modules, an ablation study was conducted under the same experimental conditions using 100 images selected from the comprehensive multi-scenario datasets. The results of U-Net, a GAN, RU-Net, and Crack-GAN are compared, as shown in Figure 9.

The baseline U-Net model demonstrated relatively low recall and F1 scores, indicating significant under-detection issues. With the introduction of the GAN framework, the model achieved a notable improvement in recall to 72.27% and an increased F1 score of 69.59%, albeit with a 19 ms increase in runtime, reflecting the computational trade-off required for adversarial training benefits. The RU-Net architecture, incorporating residual modules, maintained high pixel accuracy (PA) while substantially boosting recall to 80.42% and achieving an F1 score of 73.18%. Moreover, it exhibited superior computational efficiency compared to the original U-Net, demonstrating that residual connections effectively enhanced feature transmission. The proposed Crack-GAN achieved optimal overall performance; in terms of detection efficacy, it attained a recall of 86.67% and an F1 score of 75.69%, representing significant improvements of 44.4% and 20.0%, respectively, over the U-Net baseline. Regarding computational efficiency, the 48 ms runtime constituted a 30.4% reduction compared to the GAN framework. Additionally, its superior IoU and mAP@50 metrics validated enhanced segmentation precision and localization accuracy.

The experimental results show that by integrating the adversarial learning mechanism of the GAN and the feature optimization ability of the residual network, Crack-GAN effectively solves the problems of missed detection and false detection in traditional methods and provides a reliable solution for the accurate recognition of tunnel cracks while maintaining high operating efficiency.

### 4.5. Analysis of the Results of Different Methods

**Figure 10:**
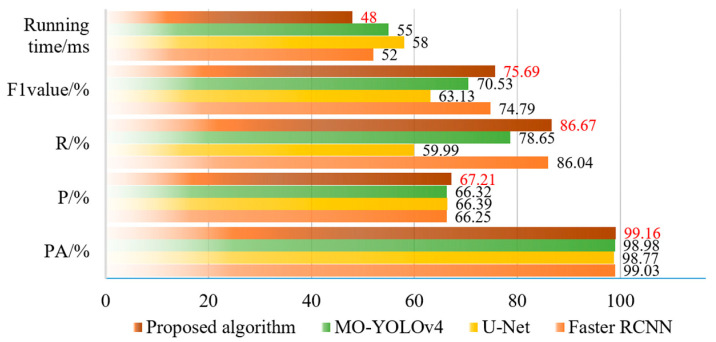
Comparative performance evaluation of different methods, and the red font is the calculation result of the algorithm proposed.

**Figure 11:**
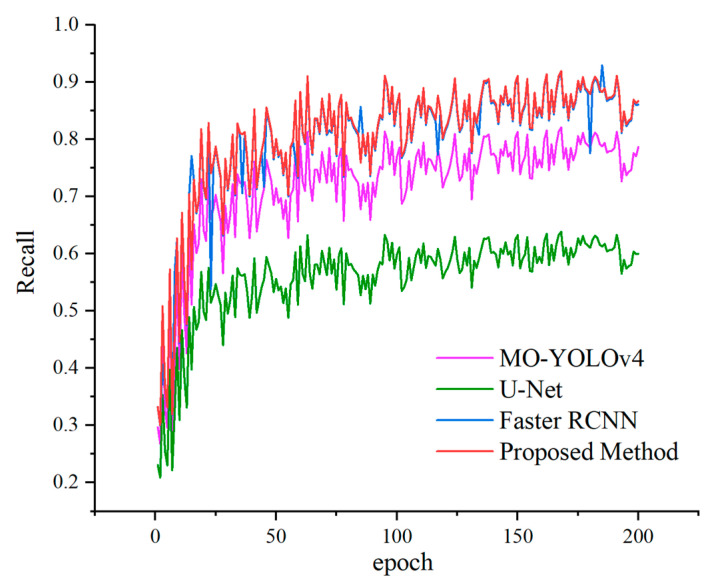
Comparative recall of different methods.

To demonstrate the segmentation performance of the proposed method, a comparative analysis was conducted through experiments. In this comparative study, the proposed method was tested alongside MO-YOLOv4 [33], U-Net, and Faster R-CNN. Recognition was performed with the 3000 images from the datasets to verify the results of the performance evaluation indicators of the improved model as shown in Figure 10 and Figure 11.

From the results of Figure 10, we can find that the proposed Crack-GAN achieved improvements in all four metrics: PA, precision (P), recall (R), and F1 score. While maintaining an F1 score comparable to that of the Faster RCNN network, it achieved a computational efficiency similar to that of the U-Net network. Specifically, the F1 score of Crack-GAN was 12.56% higher than that of U-Net, and its computation time was faster than that of Faster RCNN. Figure 11 clearly demonstrates that the proposed algorithm achieved significantly higher recall than both U-Net and MO-YOLOv4. This superior recall indicates the model’s capability to detect the majority of actual cracks, thereby ensuring the reliable recognition of critical fracture information in complex tunnel environments, a crucial capability for tunnel safety monitoring and maintenance.

**Figure 12:**
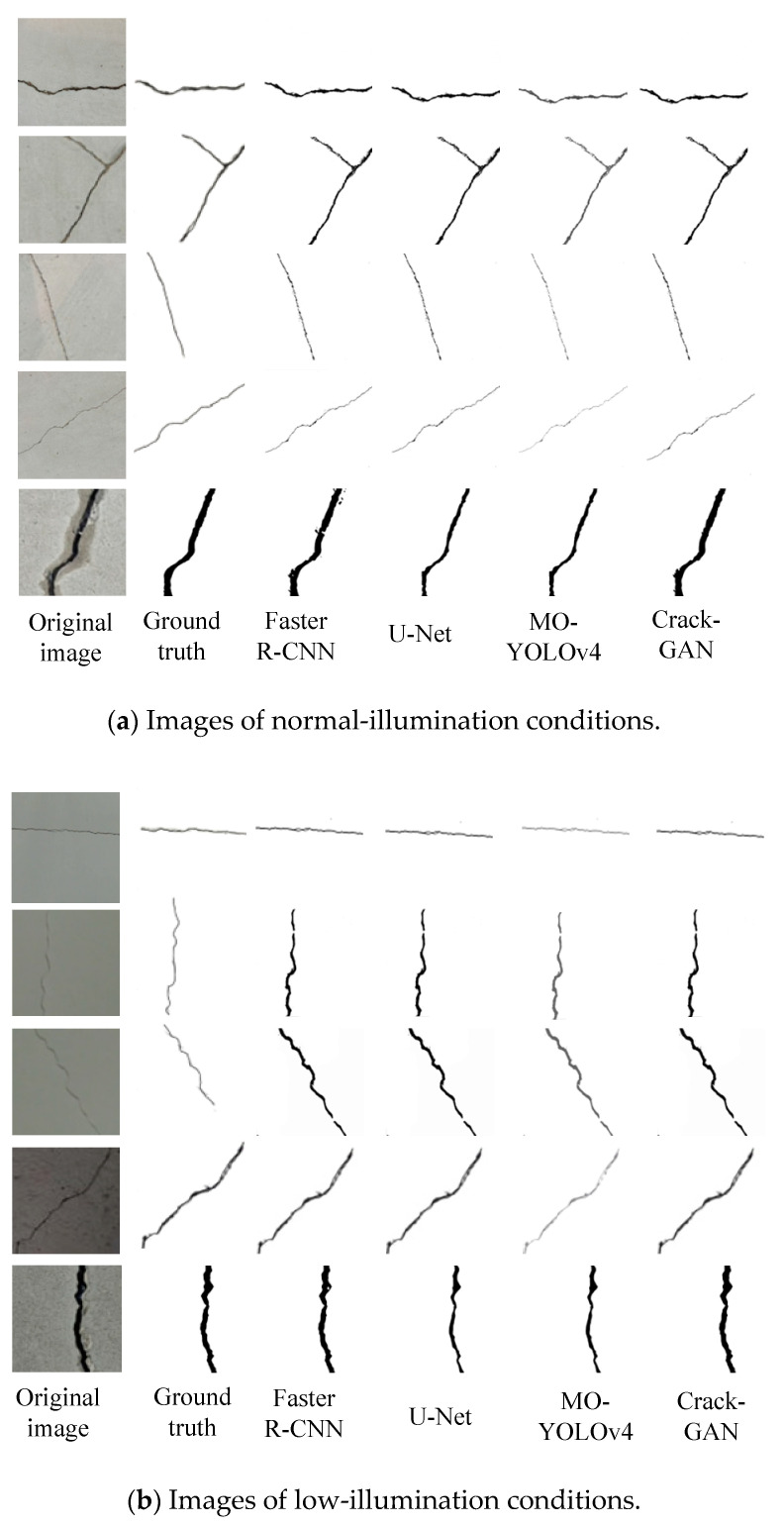

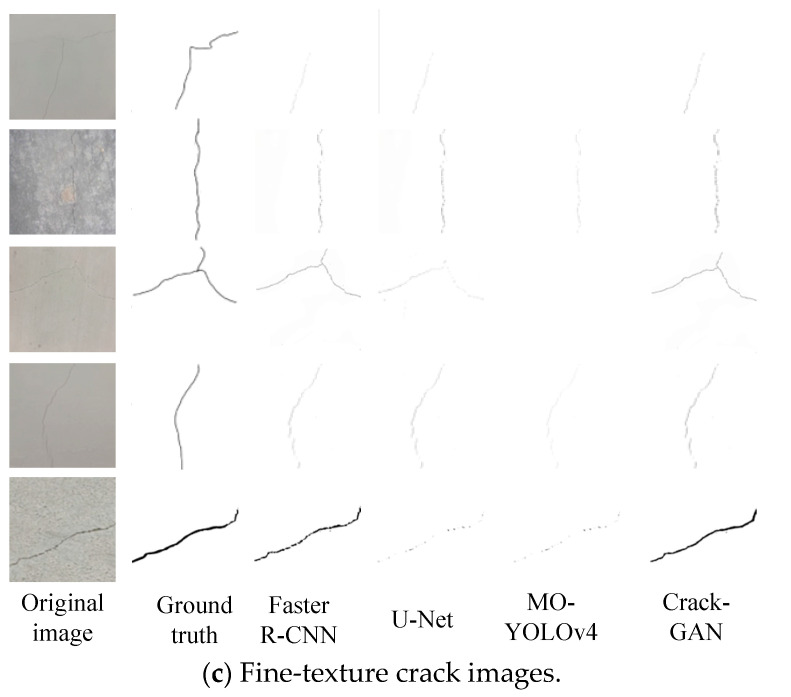
Crack segmentation results.

Five images with different crack types were randomly selected from the 3000 images from the datasets. The experimental data of crack segmentation results are shown in Figure 12.

In Figure 12, the last segmentation result in each image group represents a locally magnified output. Under normal-illumination conditions, all four models could successfully segment cracks. While YOLOv4 is primarily designed for object detection, its modified version, MO-YOLOv4, demonstrates advantages in crack detection tasks, including an enhanced multi-scale feature extraction capability and improved resistance to background interference. However, due to the typically slender and irregular nature of cracks, MO-YOLOv4’s bounding box regression may fail to accurately capture crack geometries. Although its detection precision improved under low-noise conditions, the algorithm still struggled to achieve precise crack segmentation. In contrast, both U-Net and Faster R-CNN delivered accurate segmentation results. For fine-texture crack images, MO-YOLOv4 exhibited limited detection capability, often resulting in missed or false detections. While Faster R-CNN could identify fine cracks, its segmentation performance remained inferior to that of U-Net. Crack-GAN demonstrated significant advantages in the task of tunnel crack segmentation, particularly with complex backgrounds and under noise interference. It accurately segmented cracks while avoiding misclassification and missed detection. This capability makes Crack-GAN highly effective in practical applications, as it maintains high precision while effectively handling fine-texture crack images and complex backgrounds.

## 5. Conclusions

According to the characteristics of tunnel crack images, this paper used image enhancement technology based on the SPGD algorithm to enhance the details of crack images and proposed Crack-GAN as a tunnel crack segmentation network. The model uses adversarial loss for adversarial learning in the discriminator network to reduce additional computation and improve the accuracy of crack segmentation. In the tunnel crack dataset test, the four indexes of PA, P, R, and F1 score were 99.16%, 67.21%, 86.67%, and 75.69%, respectively, and all the models achieved excellent results and had fast calculation speed. The proposed method can improve the recognition efficiency and accuracy of tunnel cracks. The research results of this paper provide reliable data support for the next steps in fracture hazard assessment.

## Figures and Tables

**Table 1:** Experimental environment.

Experimental Environment	
GPU	NVIDIA GeForce RTX 3090 Ti
Operating system	Windows 11
Programming language	Python3.7
Deep learning framework	Pytorch1.10.0

**Table 2:** Pixel evaluation category.

Item	Actual Crack Pixels	Real Non-Crack Pixels
Test crack pixels	TP	FP
Test non-crack pixels	FN	TN

## Data Availability

The data that support the findings of this study are available from the corresponding author upon reasonable request.
